# Genomic basis of symbiovar mimosae in *Rhizobium etli*

**DOI:** 10.1186/1471-2164-15-575

**Published:** 2014-07-08

**Authors:** Marco A Rogel, Patricia Bustos, Rosa I Santamaría, Víctor González, David Romero, Miguel Ángel Cevallos, Luis Lozano, Jaime Castro-Mondragón, Julio Martínez-Romero, Ernesto Ormeño-Orrillo, Esperanza Martínez-Romero

**Affiliations:** Ecological Genomics programs, Genomics Science Center, CCG, Cuernavaca, Morelos Mexico; Evolutionary Genomics programs, Genomics Science Center, CCG, Cuernavaca, Morelos Mexico; Genome Engineering programs, Genomics Science Center, CCG, Cuernavaca, Morelos Mexico

**Keywords:** Legume nodulation, Bacterial symbiosis, Nitrogen fixation, Host specificity

## Abstract

**Background:**

Symbiosis genes (*nod* and *nif*) involved in nodulation and nitrogen fixation in legumes are plasmid-borne in *Rhizobium*. Rhizobial symbiotic variants (symbiovars) with distinct host specificity would depend on the type of symbiosis plasmid. In *Rhizobium etli* or in *Rhizobium phaseoli,* symbiovar phaseoli strains have the capacity to form nodules in *Phaseolus vulgaris* while symbiovar mimosae confers a broad host range including different mimosa trees.

**Results:**

We report on the genome of *R. etli* symbiovar mimosae strain Mim1 and its comparison to that from *R. etli* symbiovar phaseoli strain CFN42. Differences were found in plasmids especially in the symbiosis plasmid, not only in *nod* gene sequences but in *nod* gene content. Differences in Nod factors deduced from the presence of *nod* genes, in secretion systems or ACC-deaminase could help explain the distinct host specificity. Genes involved in *P. vulgaris* exudate uptake were not found in symbiovar mimosae but *hup* genes (involved in hydrogen uptake) were found. Plasmid pRetCFN42a was partially contained in Mim1 and a plasmid (pRetMim1c) was found only in Mim1. Chromids were well conserved.

**Conclusions:**

The genomic differences between the two symbiovars, mimosae and phaseoli may explain different host specificity. With the genomic analysis presented, the term symbiovar is validated. Furthermore, our data support that the generalist symbiovar mimosae may be older than the specialist symbiovar phaseoli.

**Electronic supplementary material:**

The online version of this article (doi:10.1186/1471-2164-15-575) contains supplementary material, which is available to authorized users.

## Background

Bacterial nitrogen fixation in legume nodules contributes to plant nutrition and allows plants to grow in nitrogen deficient soils. Genes for plant nodulation and nitrogen fixation are plasmid-borne in *Rhizobium* spp. (reviewed in [1]) and symbiovars define host specificity. There are over twenty different symbiovars reported not only in *Rhizobium* but also in *Bradyrhizobium* and in other genera of nodule forming bacteria [2–7]. The term symbiovar was proposed as a counterpart to the term pathovar in pathogenic bacteria [2]. A theoretical model proposes that a single species may exhibit alternative symbiovars depending on the presence of symbiotic plasmids or symbiotic islands [2]. The same symbiovar may be present in distinct species as a consequence of the lateral transfer of symbiotic plasmids or islands. Symbiotic genes and other genes associated with niche adaptation may have evolutionary histories independent of the evolution of the chromosomal genes [8]. Two symbiovars are recognized in *Rhizobium etli*: phaseoli (conferring the ability to nodulate *Phaseolus vulgaris*) and mimosae (involved in nodulating mimosas and *P. vulgaris*, [9]). Symbiovar mimosae strains were isolated from *Mimosa affinis* in Morelos and have a broad host range, including plants of *M. affinis, Leucaena leucocephala*, *Calliandra grandiflora, Acaciella angustissima* as well as *P. vulgaris* [9]. Symbiovar mimosae was originally distinguished from sv. phaseoli by the sequence of a few symbiotic genes and by the organization of *nif* and common *nod* genes. Multiple copies of *nifH* genes and a *nodA* gene separated from *nodBC* found in sv. phaseoli and not in sv. mimosae served as a molecular basis to distinguish these symbiovars [10, 11]. Phylogenies indicated that symbiovar mimosae and phaseoli *nifH* genes were related [9] and similar to the corresponding gene in sv. gallicum [12]. Different origins of replication were found in sv. phaseoli and sv. mimosae symbiotic plasmids and both symbiotic plasmids were compatible [9]. It has been proposed that sv. mimosae is older than sv. phaseoli and that the phaseoli symbiotic plasmid was selected by *P. vulgaris* [13]. *P. vulgaris* is a recent species (probably two million years old [14]), while mimosas seem to be older.

Mimosas are distributed worldwide with Brazil and Mexico as main diversification sites. Mimosas in South America are nodulated by β-Proteobacteria like *Burkholderia* or other β-Proteobacteria [15–19] while mimosas in Mexico are only exceptionally nodulated by *Burkholderia* (unpublished). Mimosas from Mexico and Brazil are phylogentically separated [20]. Additionally abiotic conditions like pH and soil nitrogen content may account for their differences in symbionts [21]. Native mimosas in India are nodulated by sinorhizobia [22], that we have also found in some mimosa nodules in Mexico (unpublished).

Based on multilocus enzyme electrophoresis analysis, *M. affinis* isolate Mim1 was found to group closely to *R. etli* sv. phaseoli CFN 42 [9]. Other *M. affinis* isolates such as Mim2 were separated from CFN42 (Figure two in [9]) and thus Mim2 has been recently reassigned to *Rhizobium phaseoli* [1, 23]. Therefore we recognize now that symbiovar mimosae exists in *R. phaseoli* as well as in *R. etli*. It is the aim of this work to define the genomic differences between two *R. etli* strains (CFN42 and Mim1) representing the symbiovars phaseoli and mimosa, respectively.

## Methods

### Strains and growth conditions

*Rhizobium* strains were grown overnight at 28ºC in PY medium [24] after recovery from glycerol stocks at -80ºC. Bacterial strains to be inoculated on plants were grown on solid PY media and resuspended in water to an OD_600_ of 0.5. For PCR or DNA isolation, bacteria were grown in liquid PY cultures [24].

### Plasmid profiles

Plasmid profiles were visualized on agarose gels according to the protocol described by Hynes and McGregor [25]. Plasmid patterns from *R. etli* CFN42 or *R. leucaenae* CFN299 were used as references.

### Plant nodulation assays

*L. leucocephala* seeds were treated with concentrated sulfuric acid for 15 min, rinsed with water and surface disinfected with sodium hypochlorite as described [26]; the same procedure was used to disinfect *P. vulgaris* seeds. Seeds were germinated in water-agar plates in the dark and transferred to flasks after 3 days. *L. leucocephala* plants were grown in vermiculite flasks with N free Fahraeus nutrient solution for 40 days and *P. vulgaris* plants in agar flasks with the same nutrient solution for 14 days.

### Genome sequencing, assembly and annotation

Genomic DNA from *R. etli* Mim1 was sheared to produce two paired-end libraries for 454 pyrosequencing, one with 3 Kb inserts and the other with 8 Kb inserts. An additional 3 Kb library was sequenced only at one end. The total amount of reads were 512,236 paired reads and 112, 079 single reads. Library construction and sequencing was done at Mogene LC (St. Louis, MO, USA). Additionally two paired-end libraries were constructed, one with 200 bp inserts and the second with 2 Kb inserts. Both libraries were sequenced by Illumina at BGI (Beijing, China). A total amount of 16, 000, 000 short-paired readings (50–60 bases) were assembled. To improve the scaffolding a BAC library was constructed in pIndogo BAC-5™ vector by BIOS&T (Quebec, Canada) using fragments from a partial genomic DNA *Hind*III digestion. 105 BAC-ends were sequenced with ABI3730xl sequencer by Sanger method. Additionally, three BACs were completely sequenced with the same technology at the Center for Genomic Sciences (Cuernavaca, México). Two of these BACs were selected by hybridizing with *nifH* and other pSym probes; they embraced half of the pSym plasmid sequence. The third BAC was from the chromosome. Final assembly of the symbiotic plasmid was obtained using sequence reads from the three sources of information: BAC sequences, Illumina, and 454 pyrosequencing.

Different assembly strategies were used with the following programs: Newbler 2.5.3 (ROCHE), Velvet 1.1.06 [27], Sspace-Basic 2.0 [28], and Consed v23 [29]. ORFs were predicted with Glimmer 3 [30], and annotations were done in Artemis 12.0 graphic display [31] using previous annotations made for *R. etli* CFN42 [32] and comparing with the non-redundant data base of the Genbank [33], Interpro database [34], and IS database (http://www-is.biotoul.fr).

### Sequence analysis

Average nucleotide identity (ANI) was calculated using the JSpecies software [35]. The DNA conservation between two genomes or replicons was estimated by obtaining an alignment with NUCmer [36], run with default parameters, and dividing the summed lengths of all aligned regions by the length of the genome or replicon and expressing the value obtained as a percentage. Common and specific protein families between *R. etli* CFN42 and Mim1 were detected using MCL as described [37].

### Genomic islands

Alien Hunter v1.7 software [38] was used to analyze the chromosome sequence of *R. etli* sv. mimosae Mim1 and *R. etli* CFN42. The minimum region length for HT detection was 5 kbs. The score thresholds were 12.92 and 14.96 for Mim1 and CFN42, respectively.

### Phylogenetic analysis

Alignments were performed with MUSCLE [39] and manually verified. Maximum likelihood trees were generated with PhyML [40] with tree node support evaluated by bootstrap analysis based on 1000 pseudoreplicate datasets. Phylogenetic relationships were also assessed by Bayesian inference using MrBayes 3.1 [41]. Analyses were initiated with random starting trees, run for 2,000,000 generations and three separate analyses were executed. Markov chains were sampled every 100 generations. We discarded 25% of trees as “burn in”.

### Genome accession numbers

Sequences and annotations were deposited in the Genbank database under accession numbers CP005950 (chromosome), CP005951 (pRetMim1a), CP005952 (pRetMim1b), CP005953 (pRetMim1c), CP005954 (pRetMim1d), CP005955 (pRetMim1e) and CP005956 (pRetMim1f).

## Results and discussion

### Genome of *R. etli*symbiovar mimosae strain Mim1

**Figure 1:**
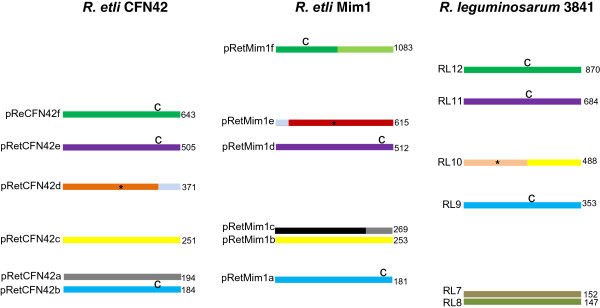
**Schematic representation of plasmid patterns of** ***Rhizobium etli*** **symbiovars phaseoli and mimosae and** ***R. leguminosarum*** **.** Equivalent replicons are indicated with the same color. *indicates the symbiotic plasmid. C indicates chromids.

The final assembly of the *R. etli* sv. mimosae Mim1 genome rendered seven circular molecules: one chromosome and six plasmids at 150× coverage on average. The chromosome was 4.8 Mb in size while the plasmids ranged in size from 181 kb to 1.08 Mb (Figure 1). Average Nucleotide Identity (ANI) and the percentage of conserved DNA between Mim1 and CFN42 were 98.6% and 82.4% respectively on a whole genome analysis, confirming that both strains belong to the same species. Lower ANI (less than 90%) was found between Mim1 and strains of other species such as *R. phaseoli* and *R. leguminosarum*.

**Figure 2:**
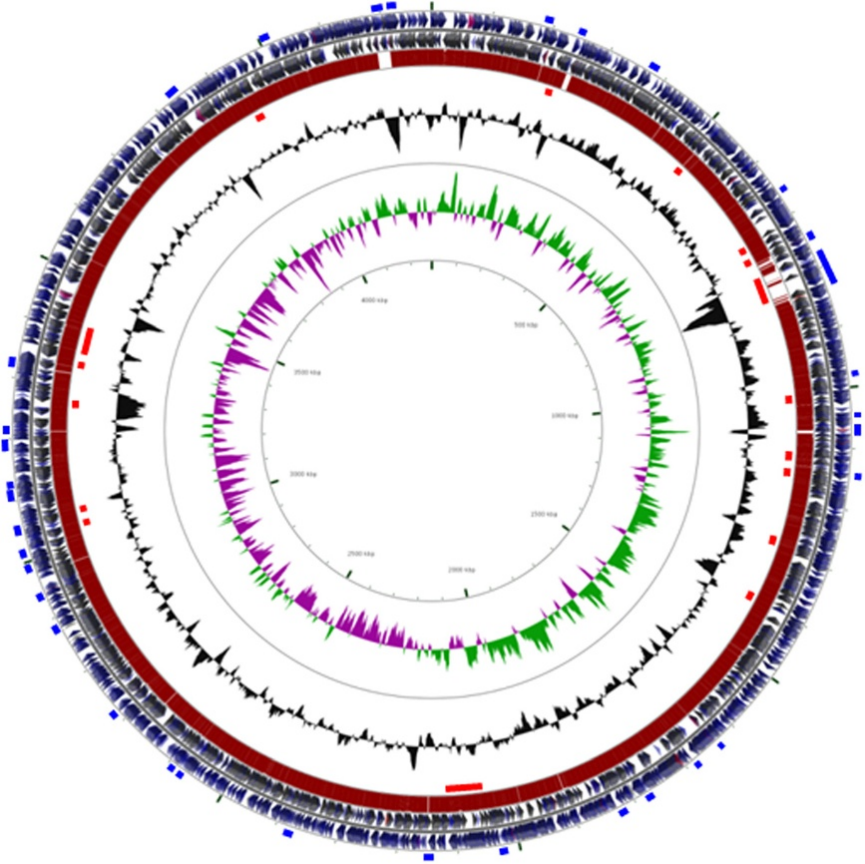
**Representation of** ***R. etli*** **Mim1 chromosome.** Circles from outermost to innermost indicate: genomic islands of Mim1 (in blue), ORFs in the leading strand, ORFs in the lagging strand, BLAST matches against CFN42 chromosome, genomic islands of CFN42 (in red), GC content, GC skew, coordinates.

There were more than twice as many unique genes in Mim1 than in CFN42, mainly in plasmids. The respective chromosomes of each strain had around 260 unique genes. In chromosomes, 35 genomic islands were identified only in Mim1 and 17 only in CFN42. (Figure 2); genes found in Mim1 genomic islands are shown in Additional file 1: Table S1. Examples of unique genes found in Mim1 and not in CFN42 are those encoding cytochrome oxidases, some chaperonins, dipeptide transporters, lactate dehydrogenase, a PHB depolymerase, a ferritin–like protein, exopolysaccharide biosynthesis genes, a type VI secretion system and menaquinone biosynthesis as well as many hypothetical genes.

**Figure 3:**
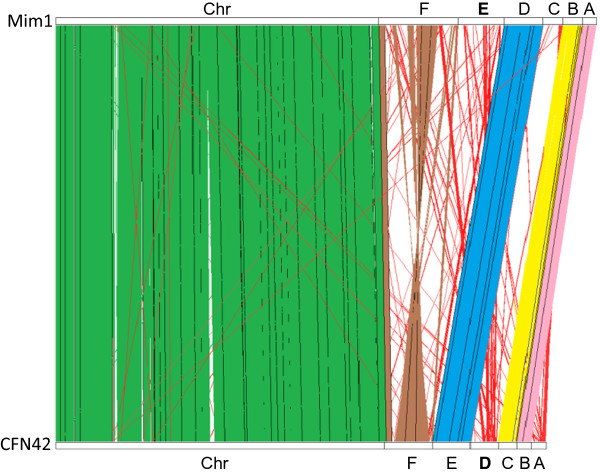
**Graphic comparison of equivalences among the** ***R. etli*** **CFN42 and** ***R. etli*** **Mim1 genomes.** Letters indicate the different extrachromosomal replicons found in both strains (see text). Letters in bold indicate the symbiotic plasmids.

**Figure 4:**
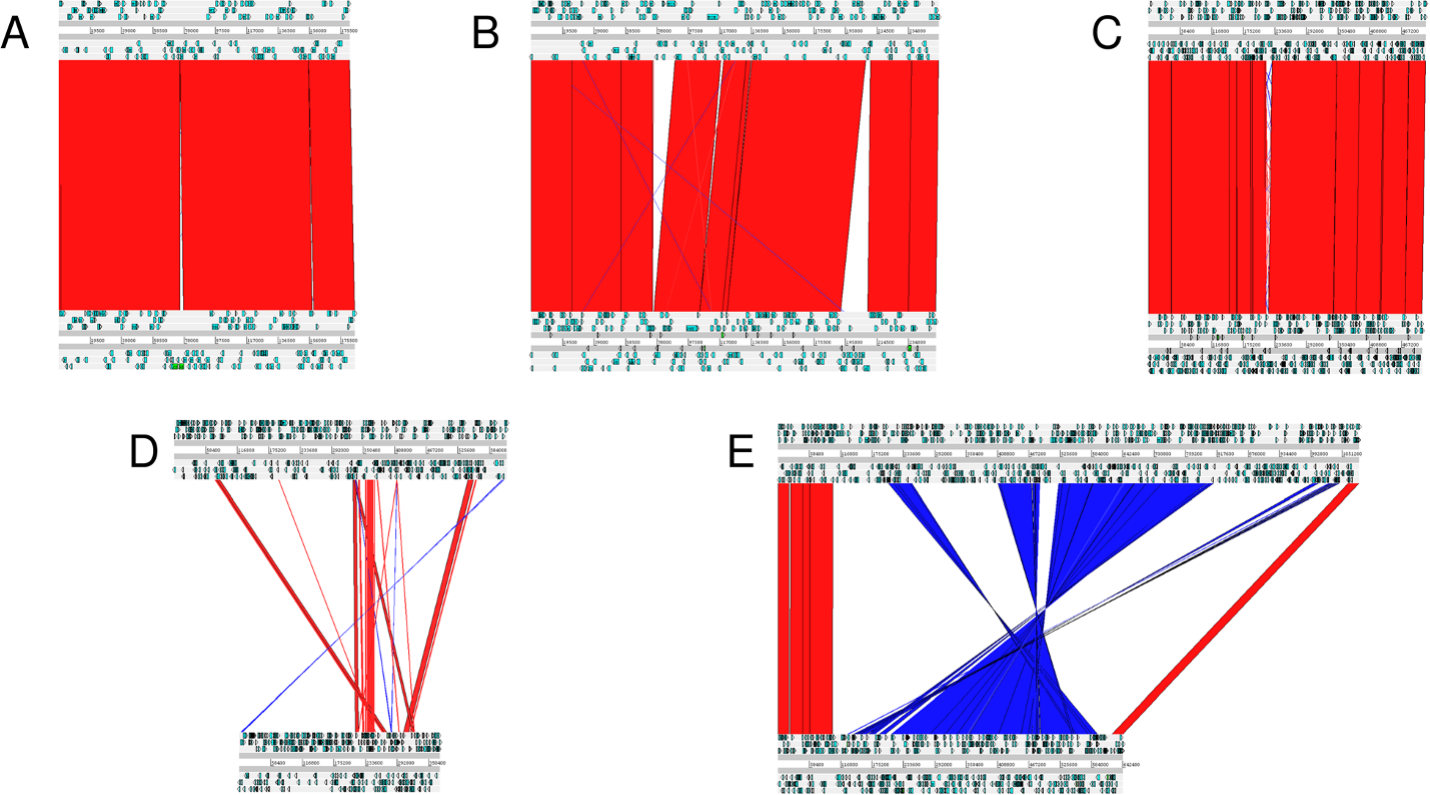
**Graphic representation of the alignments of CFN42 (top)-Mim1 (bottom) replicons. A)** pRetMim1a-pRetCFN42b, **B)** pRetMim1b-pRetCFN42c, **C)** pRetMim1d-pRetCFN42e, **D)** pRetMim1e-pRetCFN42d, **E)** pRetMim1f-pRetCFN42f. ORFs of each replicon are depicted with light blue arrows in their corresponding reading frame. Syntenic segments oriented in the same or opposite direction are joined by red and blue regions, respectively.

The conserved genome in *R. etli* strains Mim1 and CFN42 includes not only the chromosome but two extrachromosomal replicons (pRetMim1a-pRetCFN42b and pRetMim1d-pRetCFN42e) that have been designated as chromids in CFN42 [42] and one plasmid (pRetMim1b-pRetCFN42c) (Figure 3). Each of the chromids had less than 20 unique genes and the chromid pairs had an ANI around 99% (Table 1). The small replicons pReCFN42a and pRetMim1c were partially conserved and large genomic differences were found in the symbiotic plasmids (Figures 1 and 4, Table 1).

**Table 1:** **Average nucleotide identity (ANI) and conservation in percent between** ***R. etli*** **sv. mimosae Mim1 and** ***R. etli*** **sv. phaseoli CFN 42 or** ***R. leguminosarum*** **3841 replicons**

Mim1 replicons	ANI*/conservation ^§^ to the corresponding replicons in	
	***R. etli***CFN42	***R. leguminosarum***3841
pRetMim1f	97.4/51.1 (pReCFN42f)	86.8/31.4 (pRL12)
pRetMim1e (pSym)	89.2/8.8 (ReCFN42d)	
pRetMim1d	98.9/97.5 (pReCFN42e)	88.2/61.2 (pRL11)
pRetMim1c	86.4/11.4 (pReCFN42a)	
pRetMim1b	99.1/92.5 (pReCFN42c)	87.2/55.7 (pRL10)
pRetMim1a	99.2/99.9 (pReCFN42b)	87.6/58.4 (pRL9)
RetMim1Ch	99.2/97.8 (ReCFN42Ch)	88.1/78.1 (RLChr)

*Average nucleotide identity was calculated using all portions of the replicons that could be aligned with the nucmer program. These regions included both genic and intergenic regions.

^§^Percentage of the Mim1 replicon involved in the ANI calculation is expressed here as the conservation value.

Mim1 and CFN42 chromosomes were syntenic, as were the chromid pairs pRetMim1a-pRetCFN42b and pRetMim1d-pRetCFN42e, and plasmids pRetMim1b-pRetCFN42c. In comparison to *R. leguminosarum* 3841, Mim1 plasmid equivalences were similar to those reported previously for *R. etli* CFN42 [8]. ANI values were lower with *R. leguminosarum* 3841 that among the *R. etli* strains (Table 1).

All extrachromosomal replicons in Mim1 belong to the *repABC* plasmid family [43]. The protein products of the *repABC* operons of the replicon homologous pairs (pRetMim1a-pRetCFN42b, pRetMim1d-pRetCFN42e and pRetMim1b-pRetCFN42c) were almost identical with a sequence identity greater than 97.51%, strongly suggesting that members of each replicon pair belong to the same incompatibility group.

### pRetCFN42f-pRetMim1f comparison

The largest extrachromosomal replicon in Mim1 (pRetMim1f) was only partially conserved in the putative chromid pRetCFN42f (Figures 1 and 4). pRetMim1f and pRetCFN42f possess two *repABC* operons: *repABC1* and *repABC2*. The sequence identity between the two *repABC* operons in Mim1 is low. The degree of sequence identities between the corresponding *repABC* genes of pRetCFN42f and pRetMim1f is large enough to suggest that both plasmids share the same incompatibility group and evolutionary origin. However only 51% of the pRetMim1f replicon is conserved in CFN42 while 86% of pRetCFN42f was found in pRetMim1f (Figures 1 and 4).

Type VI secretion system genes were only found in the megaplasmid pRetMim1f and the conserved *impB* component is phylogenetically related to the corresponding genes in *Rhizobium* sp. KIM5 (corresponding to PEL1 lineage, [44]) and *R. leguminosarum* strain 3841. Mim1 pRetMim1f has a duplicated citrate synthase gene as in sv. tropici symbiotic plasmids. The plasmid duplicated citrate synthase gene in sv. tropici is required for eliciting a normal number of nodules and is regulated differently than the copy in the chromosome [45, 46]. A plasmid borne citrate synthase was not found in CFN42. Phylogenetic analysis of citrate synthase genes showed that the Mim1 chromosomal gene product is identical to that of CFN42, while the gene in pRetMim1f has a novel sequence only distantly related to a plasmid copy of *R. gallicum* R602 (55% identity).

Genes such as *raiI* and *raiR* were found in the conserved region of pRetCFN42f and pRetMim1f. RaiI produces homoserine lactones and RaiR is the transcriptional regulator. The *rai* system in *R. phaseoli* sv. phaseoli CNPAF 512 affects nodule number but not nitrogen fixation in *P. vulgaris* [47]. This system also controls growth in *R. phaseoli* [48] and if this is the case in *R. etli*, this would explain its conservation in both symbiovars.

A transcriptomic study compared the genome expression of *R. phaseoli* Ch24-10 in maize and *P. vulgaris* rhizospheres [49]. Over 50% of the extrachromosomal genes highly expressed in *P. vulgaris* but not in maize roots were found in a Ch24-10 replicon equivalent to pRetCFN42f. It seems that genes in this replicon are involved in plant specific interactions.

### Symbiosis plasmid gene comparison

Large differences were observed between the symbiotic plasmids of CFN42 and Mim1 (Figure 4). Around 10% and 15% of the symbiotic plasmids of Mim1 and CFN42 had conserved syntenic regions with an average nucleotide identity of 89.2% (Table 1).

Differences in symbiosis genes in CFN42 and Mim1 genomes are shown in Table 2. The Nod factor from *R. etli* sv. phaseoli strain CFN42 is a pentamer of *N*-acetylglucosamine with an acetyl fucose at the reducing end and methyl and carbamoyl groups at the non reducing end [50]. The heterologous expression in *Azorhizobium caulinodans* of Nod modification genes showed that fucosylated Nod factors were the most suitable to induce *P. vulgaris* nodulation [51]. In symbiovar mimosae no genes related to Nod factor fucosylation (*nodZ*) were observed (Table 2), in their place, *nodHPQ* genes that modify the Nod factor with sulfate, were found. Such genes are present in sv. tropici strains that are also *Leucaena* symbionts [10]. Like *R. etli* strain CFN42, Mim1 may produce nodulation factors bearing carbamoyl groups at their non reducing end residues but the position of these decorations must differ because their pSyms encoded distinct carbamoyl transferases, NolO in CFN42 and NodU in Mim1. Carbamoylation at the C-6 position introduced by NodU maybe promotes *Leucaena* nodulation [52, 53]. Both Mim1 and CFN42 symbiotic plasmids carry *nodS* involved in methylation at the non reducing end, a decoration that is essential for bean and *Leucaena* nodulation in *R. tropici* CIAT 899 [52]. A *nodO* homologue, 70% identical to that of *Rhizobium* sp. BR816, was found only in the sv. mimosae pSym. It has been shown that heterologous expression of *nodO* can improve nodulation of *L. leucocephala* by different rhizobia and can even extend the host range [54, 55].

**Table 2:** **Relevant symbiotic plasmid differences between** ***R. etli*** **sv. mimosae Mim1 and** ***R. etli*** **sv. phaseoli CFN 42**

Gene*	symbiovar	
	mimosae	phaseoli
*nodHPQ*	+	-
*nodZ*	-	+
*nolL*	-	+
*nodO*	+	-
*nolO*	-	+
*nodU*	+	-
*fixKL*	+	-
*fxkR*	+	-
*acdS*	+	-
*teu*	-	+
*hup-hyp*	+	-

*Functions of each gene are explained in the text.

Mim1 *nod* gene phylogenies are congruent, resembling the corresponding genes from sv. *giardinii* (not shown) while Mim1 *nifH* genes resemble those from sv. phaseoli. Different NodDs in phaseoli and mimosae symbiovars may reflect their affinities for the different flavonoids exuded by the different host plants. Mim1 *nodH* gene (encoding the sulfotransferase involved in the synthesis of the Nod factor) resembles the corresponding gene in *Rhizobium* sp. IE4771 that represents a novel genomic lineage related to *R. etli* and *R. phaseoli* [1, 44].

*acdS* gene coding ACC-deaminase was found in the symbiotic plasmid of Mim1 but not in CFN42. ACC-deaminase decreases the amount of ACC that is a precursor of ethylene that may diminish nodule number. A heterologous ACC-deaminase in *Rhizobium* sp TAL 1145 enhanced nodulation in *Leucaena* [56].

In CFN42, the *fixGHIS-fixNOQP* genes required for biosynthesis of the symbiotic terminal oxidase are present in the pSym and also in pRetCFN42f [57]. The regulatory genes *fixK* and *fixL* are adjacent to this reiteration in pRetCFN42f while a *fixK* pseudogene is found in the pSym [58]. In Mim1, we found that the symbiotic terminal oxidase genes are also reiterated in pRetMim1f but, in contrast to CFN42, the sv mimosae pSym carried complete *fixK* and *fixL* genes. The *fixGHIS-fixNOQP*-*fixKL* region shared by pSym and pRetMim1f plasmids in Mim1 is 95% identical while the reiterated regions in CFN42 are only 87% identical. The recently described *fxkR* gene in pRetCFN42f [59], coding for a response regulator that acts in conjunction with FixL and FixK, is present in the sv. mimosae pSym. There is a reiteration of this gene in pRetMim1f as well.

Genes involved in *P. vulgaris* exudate uptake (*teu* genes, [60]) are found in the symbiotic plasmids in symbiovar phaseoli strains *R. etli* CFN42 and *R. phaseoli* CIAT652 but they were not found in the Mim1 genome. *Rhizobium* mutants in *teu* genes had reduced nodulation competitiveness in *P. vulgaris* [60].

The symbiosis plasmid of Mim1 has genes for a type III secretion system (T3SS) that are more closely related to those found in *Mesorhizobium* and *Sinorhizobium* strains than to those encoded in the CFN42 pSym (not shown). This difference may contribute to the disparate host ranges displayed by sv. phaseoli and mimosae strains considering the function of rhizobial T3SS in specificity [61].

A cluster of *hup*-*hyp* genes encoding the components of an uptake hydrogenase (Hup) was found in the pSym of Mim1 but not in CFN42. The Mim1 products showed high identities (>70%) to their counterparts coded in *R. leguminosarum* and *R. tropici* [10, 62]. *R. tropici* CIAT 899 lacks several *hup* genes and displays a Hup minus phenotype. In Mim1, all genes except *hupE* are present. HupE is an uptake transporter for nickel [63], a metal required for Hup function. Since another nickel transporter is encoded elsewhere in Mim1 symbiotic plasmid, the Hup system could be functional.

Mim1 symbiotic plasmid has a *repABC* origin of replication as well as a *repC* gene that are not phylogenetically related to the *R. etli* CFN42 corresponding genes. pRetMim1e *repC* resembles those from *R. gallicum* and *Rhizobium* sp. sv. giardinii IE4771 (corresponding to PEL1 lineage). Mim1 *repABC* from the pSym resembles the corresponding genes in *R. endophyticum* CCGE502 but an extensive plasmid conservation was not observed. CCGE502 is Nod^-^ and does not have a symbiotic plasmid [64].

The analysis of insertion sequences in *R. etli* CFN42 suggested that the symbiotic plasmid did not significantly share IS sequences with the rest of the genome [65]. This was interpreted as evidence that the pSym was a recent acquisition in this bacterium. The analysis of IS sequences in Mim1 indicated that the symbiotic plasmid had the largest number of IS sequences, some of them shared with the chromosome, pRetMim1f and pRetMim1b, this may perhaps indicate that this symbiotic plasmid has an older history with the *R. etli* genomic background than the phaseoli plasmid. Mim1 has a large number of IS66 that are common in rhizobia.

**Figure 5:**
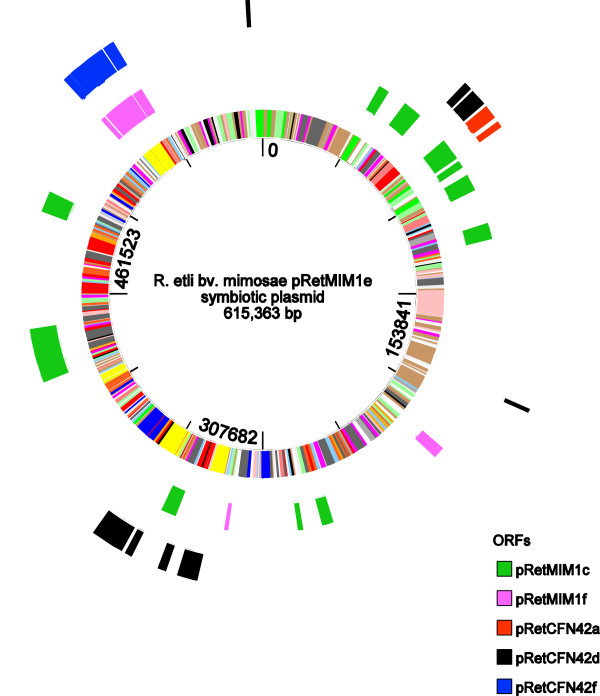
**Graphic comparison of the Mim1 symbiotic plasmid pRetMim1e compared to other rhizobial replicons.**

Genome similarities were found among the different Mim1 replicons. pRetMim1e (pSym) has similar sequences to pRetMim1c and the same is observed among pRetMim1e (pSym) and pRetMim1f (Figure 5). The similarities of Mim1 symbiotic plasmid and the putative chromid pRetMim1f could support that symbiovar mimosae symbiotic plasmid is ancestral in *R. etli.* In contrast the phaseoli symbiotic plasmid was found dissimilar to the rest of the genomic background in *R. etli*, except to pReCFN42a [32, 66].

In *R. etli* CFN42, the symbiotic plasmid and pRetCFN42a have common sequences such as *tra* and *vir* genes and repeated sequences that mediate the natural cointegration of both plasmids for the conjugative transfer of CFN42 pSym [67]. In contrast to pReCFN42a, pRetMim1c from Mim1 does not seem to participate in the transfer of the Mim1 symbiotic plasmid, which we have been unable to transfer to other bacterial hosts (Marco A. Rogel, unpublished observations).

### Plant-interaction genes not in the symbiosis plasmid

Some genes involved in plant interactions were found conserved in both symbiovars, such as those encoding Rmr extrusion pumps that may be involved in eliminating plant produced phytoalexins. *R. etli* mutants in these genes had reduced nodulation [68]. Those genes are encoded in chromid pRetCFN42b in CFN42 and in the corresponding replicon pRetMim1a. *rmrA* gene had 97% identity and *rmrB* and *rmrR* genes 98% in CFN42 and Mim1 genomes. Homologous genes were found being expressed in different plant rhizospheres [1, 49, 69].

Even though sv. mimosae strains are capable of forming nodules in *Leucaena*, we did not find genes resembling *mid* or *pyd* genes involved in the catabolism of the toxic amino acid derivative mimosine found in *Leucaena* plants. Such genes were described from a *Rhizobium* sp. strain TAL 1145 (related to *R. tropici*) that was isolated from *Leucaena* plants [70]. *Mimosa pigra* has a much lower level of mimosine that *Leucaena* plants [71] and data for other mimosa species is not available.

### Symbiovar phaseoli is prevalent in different *Phaseolus vulgaris*nodule bacteria

*R. etli* sv. mimosae strains have a broader host range than symbiovar phaseoli strains. In particular, *L. leucocephala* plants served as a host to distinguish symbiovar mimosae strains. Thirty-six *P. vulgaris* nodulating bacteria (with *R. etli*-like 16S rRNA gene sequences) obtained from the rain forest of Los Tuxtlas in Mexico corresponded to symbiovar phaseoli on the basis of *nodA* gene organization [10] and for being unable to nodulate *Leucaena* plants. We found that some strains that were previously classified as *R. etli* such as CIAT652, Ch24-10, CNPAF512, 8C-3 and Brasil5 now assigned to *R. phaseoli* [1], as well as others like Kim5, GR56 and CIAT 894 now assigned to recently named novel lineages [44] corresponded to sv. phaseoli when we analyzed their genomes. Symbiotic plasmids are highly conserved in symbiovar phaseoli [66] perhaps from being recently evolved [13]. Considering that the majority of *P. vulgaris* nodule isolates tested corresponded to sv. phaseoli we may conclude that this symbiovar is better adapted to its host, thus phaseoli seems to be a specialist symbiovar having a narrow range not including mimosa plants. The phaseoli symbiovar is found in several *Rhizobium* species or lineages (*R. gallicum, R. giardinii, R, phaseoli, R. etli* and *Rhizobium* sp. corresponding to PEL1 lineage). The widespread of this symbiovar may be in relation to its host historic worldwide distribution and to the transferability of the symbiotic plasmid, seemingly an epidemic plasmid. Besides having the phaseoli symbiovar, the *Rhizobium* species mentioned above have additional generalist symbiovars: gallicum or giardinii or mimosae Having alternative symbiovars with different host ranges may be advantageous in rhizobia, as it expands their legume niches and allows them to avoid the specialist-generalist dilemma.

## Conclusions

The term symbiovar is validated with genomic analyses that show that a common genomic background may harbor different symbiotic plasmids determining host specificity. However, besides differences in the symbiotic plasmids there were differences in other ERs and in the chromosomes in the two strains analyzed, CFN42 and Mim1. In Mim1, Nod factors with sulfate modifications, secretion systems or ACC-deaminase may help explain the extended host range of symbiovar mimosae. In CFN42, *teu* genes that participate in exudate uptake [60] and genes involved in Nod factor fucosylation (*nodZ*) may contribute to *P. vulgaris* host specialization. The discussion that mimosae is older than phaseoli may apply to gallicum and giardinii, thus we propose that gallicum and giardinii are older than phaseoli.

## Electronic supplementary material

Additional file 1: Table S1: Genes found in different genomic islands exclusive of Mim1. (XLS 104 KB)
